# Sublingual immunotherapy in children: facts and needs

**DOI:** 10.1186/1824-7288-35-31

**Published:** 2009-10-23

**Authors:** Gian Luigi Marseglia, Cristoforo Incorvaia, Mario La Rosa, Franco Frati, Francesco Marcucci

**Affiliations:** 1 Department of Paediatrics - Foundations IRCCS Policlinico San Matteo, University of Pavia, Italy; 2 Allergy/Pulmonary rehabilitation, ICP Hospital, Milan, Italy; 3 Department of Pediatrics University of Catania, Italy; 4 Pediatrics, University Department of Medical and Surgical Specialties and Public Health, Perugia, Italy; 5 Medical and Scientific Department, Stallergenes, Milan, Italy

## Abstract

Allergen specific immunotherapy (SIT) is the practice of administering gradually increasing doses of the specific causative allergen to reduce the clinical reactivity of allergic subjects, and is the only treatment targeting the causes of hypersensitivity and not only the symptoms, as done by drugs. The traditional, subcutaneous immunotherapy (SCIT) was burdened by the problem of systemic reactions which may be sometimes severe and - though very rarely - even fatal. This was the background to develop non injections routes for SIT and particularly sublingual immunotherapy (SLIT), that emerged as a real treatment option for respiratory allergy.

A number of studies was conducted to evaluate efficacy and safety of SLIT, the first meta-analysis - including 22 placebo-controlled trials - concluded for positive results in both issues, but the number of studies on children was too low to draw definite conclusions. Since then, many other studies became available and make possible to analyze SLIT in children in its well defined aspects as well as in sides still requiring more solid data.

## Introduction

Allergen specific immunotherapy (SIT) is the practice of administering gradually increasing doses of the specific causative allergen to reduce the clinical reactivity of allergic subjects, and is the only treatment targeting the causes of hypersensitivity and not only the symptoms, as done by drugs [1]. The traditional, subcutaneous immunotherapy (SCIT) was burdened by the problem of systemic reactions which may be sometimes severe and - though very rarely - even fatal [2]. This was the background to develop non injections routes for SIT and particularly sublingual immunotherapy (SLIT), that emerged as a real treatment option for respiratory allergy [3].

A number of studies was conducted to evaluate efficacy and safety of SLIT, the first meta-analysis - including 22 placebo-controlled trials - concluded for positive results in both issues, but the number of studies on children was too low to draw definite conclusions [4]. Since then, many other studies became available and make possible to analyze SLIT in children in its well defined aspects as well as in sides still requiring more solid data.

## Efficacy of Slit in Children

The clinically efficacy of SLIT, as of SIT in general, is evaluated by the decrease in symptom scores of rhinitis and asthma and in consumption of symptomatic drugs. Many placebo-controlled studies are conducted on small patient populations and cannot achieve a reliable statistical significance, but their combined evaluation by the tool of meta-analysis is considered an adequate method to obtain more robust data [5]. The results obtained by the Cochrane Collaboration method [6] are expressed as standardized mean difference (SMD) and allow to compare the effect of SLIT on actively and placebo treated patients. Also systematic reviews, that is, literature analysis without using the Cochrane method, are available.

The progressive increase in number of SLIT studies addressing the pediatric population made possible to perform specific meta-analyses and systematic reviews. The first systematic review included the studies up to June 2003, which were evaluated qualitatively, and concluded for low to moderate efficacy of SLIT only in children with house dust mite induced mild to moderate asthma [7]. A meta-analysis by Olaguibel et al including 7 randomized controlled studies conducted on children aged up to 14 years was substantially in agreement, since it found that SLIT was significantly effective on asthma symptoms (SMD -1.42, p = 0.01) and on drug consumption (SMD -1.01, p = 0.06), while the improvement did not reach the significance for nasal and conjunctival symptoms [8].

A further meta-analysis on SLIT in children was published in 2006 [9]: in this case the evaluation concerned the efficacy on allergic rhinitis including 10 randomized controlled studies, with an overall number of 484 patients (245 actively and 239 placebo treated). A significant reduction of both symptoms (SMD - 0.56, p = 0.02) and medication (- 0.76, p = 0.03) was observed. A notable aspect was provided from the sub-analysis addressing the length of treatment and the kind of allergen administered, which demonstrated a higher efficacy for durations longer than 18 months and for pollen allergens compared to house dust mites.

The same group performed a meta-analysis on the efficacy of SLIT in allergic asthma, analyzing 9 studies on pediatric patients which included a total number of 441 patients, 232 actively treated and 209 placebo-treated [10]. A significant reduction was found in both symptoms scores (SMD - 1.14, p = 0.02) and drug use (SMD -1.63, p = 0.007).

A systematic review in the same year by Roder et al evaluating any form of immunotherapy in children concluded for no evidence of effectiveness in the subgroup of 11 studies on SLIT, but the review was based on analysis of each single study and not on pooling all data together [11]. The authors justified such approach with the relevant heterogeneity of the available studies.

Actually, heterogeneity, which is mainly due to different scoring systems in the various studies. is a limit of meta-analysis. However, a data source alternative to meta-analysis are studies conducted on large numbers of patients that provide adequate statistical power. The recent preparations for SLIT in orosoluble tablet of grass pollen extract were evaluated on large populations, including 253 children treated by a one grass (Phleum pratense) extract [12], and 278 children treated with a 5-grass pollen extract [13]. These studies showed a highly significant improvement in symptom and rescue medications scores in actively treated compared with placebo treated patients during the grass pollen season. Thus, the criticism on the efficacy of SLIT in children does not seem to have ground. An updated and balanced review on this issue by Larenas-Linnemann was recently published [14]. The author after accurate analysis of all the available studies concluded that "evidence of effect is confirmed for SLIT in children with allergic rhinitis or asthma caused by pollen exposure", while there is yet room for investigations on long-term effects and preventive action of SLIT, as well as on optimal dosing for dust mites. Indeed, the dosing is a pivotal factor, and the dose-dependence of efficacy in children treated with pollen extract was clearly demonstrated both clinically [15] and immunologically [16]. Dose-response studies in mite allergic children are warranted. Another important observation concerns the capacity of SLIT to prevent the development of asthma in children with seasonal rhinitis treated with grass pollen extract compared with subjects treated with standard symptomatic drugs [17].

## Safety of Slit in Children

All systematic revisions and meta-analysis found that the most common adverse events to SLIT, regardless the age, are local reactions in the oropharynx - with itching, tingling and swelling in the mouth - followed by local gastrointestinal reactions - with nausea, vomiting or diarrhea - and that systemic reactions such as asthma, rhinitis, or urticaria, are quite rare [4,18-20]. An increased risk of systemic reactions is apparent in subjects undergoing SLIT because of previous systemic reactions to SCIT [21,22]. In particular, one of the cases of anaphylaxis concerned a pediatric patient, who had had urticaria to previous SCIT treatment and developed an anaphylactic reaction after the very first dose of a grass pollen tablet formulation with no updosing phase [21].

Some studies addressed specific safety issues in children. SLIT was well tolerated using ultra-rush schedules - that reach the maintenance dose in a few hours [23] - and also starting the treatment during the pollen season [24]. Two studies demonstrated that SLIT is safe also in children younger than 5 years (that is the age limit indicated for SCIT), as assessed by comparable rate and kind of adverse effects in patients aged less or more than 5 years [25,26]. A further observation regarded children treated with one or multiple allergen extracts, who showed comparable rates of side effects, more than 90% being mild and self-resolving [27].

Concerning SLIT with house dust mites extracts, a recent study (including both adults and children) reported a comparable safety and tolerability in patients treated continuously or intermittently, i.e. 2-month treatment alternate to-2 month suspension [28].

## Issues Influencing the Feasibility of Slit

As stated above, SLIT has clear evidence of efficacy and safety. Still, there are aspects influencing its clinical results, such as the compliance, or its prescription, such as the cost-effectiveness.

### Compliance to SLIT

According to established definitions, compliance is "The extent to which a patient's behavior matches the prescriber's advice" and adherence is "The extent to which the patient's behavior matches agreed recommendations from the prescriber" [29], and both of them are essential for the clinical outcome of a medical treatment. A number of studies conducted on SCIT showed that the major cause of noncompliance was the inconvenience, related to injections and particularly to their frequency, and the cost of the treatment [30]. SLIT has different compliance issues than SCIT, because it is administered at home by patients themselves and thus it is not affected by most causes reported for non-compliance to allergen injections, having instead compliance problems similar to drug treatment. Some studies not specifically designed for compliance (for instance safety and tolerability analyses) reported that treatment withdrawal is frequently caused by repeated local reactions in the mouth or at gastrointestinal level [4,18]. Concerning specific compliance and adherence studies, the available data indicate quite satisfactory results.

In a study on children treated with SLIT by an allergen extract in monodoses, parents were interviewed by unscheduled phone calls at the third and sixth month of SLIT and asked to count at once the remaining doses; a compliance rate higher than 75% was found in 85% of children at the third month and in 84% of children at the sixth month; the major cause of withdrawal (5.6% of cases) was the cost of treatment, while side effects accounted for 1.4% of stopping [31]. In a study comparing compliance to SLIT, SCIT and local nasal immunotherapy in children, data on SLIT concerned 806 patients, 173 of whom (21.4%) were noncompliant, with a highly significant difference (p < 0.0001) for a better compliance in hospital setting (90.5%) compared to private office setting (61.2%); the most common reason of withdrawal was the cost of treatment, reported globally in 36.4% of cases, followed by inconvenience, feeling of inefficacy, and side effects [32].

### Cost-effectiveness of SLIT

Many studies are available, recently reviewed, showing that SLIT provides economic advantage compared with drug treatment by bringing a better clinical outcome at a reduced cost or an extra benefit at a very acceptable extra cost [33]. Concerning children, fhe first published study dealt with the evaluation of cost effectiveness of SLIT, with the high dose suggested in the ARIA document [34], in subjects with allergic rhinitis and asthma. From records of pediatric patients seen for respiratory allergy, who had 1-year data prior to receive SLIT and 3-year data on high dose SLIT, outcome measures (the number of exacerbations, visits, absence from nursery or school) were analyzed. Moreover, direct costs (Euro spent on drugs, specialists visits, and SLIT) and indirect costs (costs resulting from children school and parental work loss) were considered. A second analysis compared a sub-group of children with allergic asthma, using a control group for costs, based on records of patients not treated with SLIT, extracted from a network-database of pediatricians. An overall number of 135 children were analyzed, 46 with perennial and 89 with seasonal allergy, with comparable gender and age distribution. A substantial reduction was found in all outcome measures during SLIT compared with the previous period. The average annual cost/patient was Euro 2672 before SLIT initiation and Euro 629/year during SLIT. Similar results were found for allergen subgroups. The asthma analysis involved 41 children with SLIT and 35 controls, and also showed a substantial reduction in outcomes, though the direct cost per patient over the 4 years follow-up was € 1182 for SLIT-treated children and € 1100 for controls [35]. A study conducted in France estimated that in children treated with SLIT for house dust mite or pollen allergies, the incremental costs per asthma case avoided over a 7-year period, compared with standard symptomatic treatment, were 3938 Euro for dust mite and 824 Euro for pollen allergy; of note, there was an economic advantage of SLIT also versus SCIT for pollen allergy, since the incremental cost for the latter was 1708 Euro [36].

## Conclusion

SLIT was successfully introduced in Europe mainly on safety grounds and in some countries, including Italy, is currently more frequently employed than SCIT. The analysis of the abundant literature supports the use of SLIT in children with rhinitis and asthma caused by sensitization to seasonal allergens, while further studies are needed to demonstrate a full effectiveness in sensitization to perennial allergens. Favourable data obtained from studies on compliance and cost-effectiveness make SLIT a feasible treatment for treatment with respiratory allergy.

## Competing interests

The authors declare that they have no competing interests.

## Authors' contributions

All author participated equally in reviewing the literaure, discussing data and drafting the manuscript. All authors read and approved the final manuscript.
